# Dose-Dependent Effects of Common Antibiotics Used to Treat *Staphylococcus aureus* on Biofilm Formation

**Published:** 2017-10-01

**Authors:** Ali Majidpour, Sara Fathizadeh, Mastaneh Afshar, Mohammad Rahbar, Mina Boustanshenas, Marjan Heidarzadeh, Leila Arbabi, Somayeh Soleymanzadeh Moghadam

**Affiliations:** 1 *Antimicrobial Resistance Research Center, Institute of immunology and infectious diseases, Iran University of Medical Sciences, Tehran, Iran*; 2 *Dept. of Microbiology, Reference Health Laboratories Research Centre, Ministry of Health and Medical Education, Tehran, Iran*; 3 *Dept. of biology, faculty of food industry and agriculture standard research institute(SRI),Karaj, Iran*; 4 *Dept. of Infectious Diseases And Tropical Medicine, School of Medicine, Iran University of Medical Sciences, Tehran, Iran*

**Keywords:** Antibiotics, *Staphylococcus aureus*, Biofilms, Minimum Inhibitory Concentrations, Microplate Assays, Therapeutic uses

## Abstract

**Background & Objective:**

*Staphylococcus aureus, *especially methicillin-resistant *S. aureus* (MRSA), represent serious nosocomial and community infections. Biofilm formation as an important virulence factor may be affected by sub-inhibitory levels of antibiotics. Few studies examined the effects of all therapeutic antimicrobial agents on clinical *S.aureus. *The current study aimed at observing the inducing and reducing effects of antibiotics, commonly used to treat staphylococcal infections on the production of staphylococcal biofilm.

**Methods:**

Four MRSA (1ATCC and 3 clinical) and 1 methicillin-susceptible *Staphylococcus aureus *(MSSA) strains with biofilm forming ability, evaluated by the Congo red agar (CRA) plate test, were employed. Biofilm formation was measured by crystal violet microtiter plate assay. Cefazolin, rifampicin, vancomycin, oxacillin, clindamycin, cotrimoxazole, minocycline, linezolid, azithromycin, and clarithromycin were added to wells ranging from 0.06to 128 µg/mL (1× to 1/1024 MIC dependent on the MIC value of each strain).

**Results:**

The current study showed that azithromycin and vancomycin had a significant inducing effect on biofilm formation. In contrast, linezolid, cefazolin, and clarithromycin, and in the second place, clindamycin and minocycline could inhibit the level of biofilm production in the sub-minimal inhibitory concentrations.

**Conclusion:**

The findings demonstrated that the biofilm formation as an important virulence factor may be affected by the subinhibitory levels of antibiotics.

## Introduction

*Staphylococcus aureus (S. aureus) *is known as a normal human skin flora and continues to be an important pathogen, particularly in nosocomial infections (1-3).The most multidrug resistant type is methicillin-resistant *S.aureus* (MRSA), a leading cause of life threatening infections (3-5). The increasing trend of antibiotic resistance, along with the capacity of biofilm formation on medical devices and tissue may cause the additional antibiotic resistance and further treatment fails(6). Biofilms are the population of microorganisms with the ability to grow on biotic and abiotic surfaces by the production of polysaccharide intercellular adhesion (PIA) encoded by *ica *ADBC(6-8). While some antibiotics are commonly used to treat staphylococcal infections, they are ineffective on them, but these therapeutic antibiotics may be capable of impressing the biofilm formation in sub-minimal inhibitory concentrations and further elimination or development of infections depends on reducing or inducing effect of these levels. The exposure of bacteria to low levels of antibiotic concentrations may take place for different reasons such as bioavailability reduction of antibiotics in systemic circulation(9), systemic absorption of topically applied antibiotics, and application of antibiotics as a growth promoters(10) may lead to find subinhibitory concentrations of these antibiotics. In some cases, even the existence of the biofilm may decrease the penetration of antibiotics to the internal milieu of biofilms and are consequently exposed to the lower levels of antibiotics due to diffusion gradients(11, 12).In addition, the wide range of antibiotics with low doses , as growth promoters in agriculture, put the organisms in exposure to low dose of drugs(10). Therefore, studying the effect of clinical antibiotics prescribed routinely by physicians for staphylococcal infections, on the biofilm formation seems necessary to improve the treatment process. The current study aimed at determined to the inducing and reducing effects of common antibiotics in sub-minimal inhibitory concentrations, on MRSA biofilm formation.

## Materials and methods

**Bacterial isolates**

Twenty-eight clinical isolates of *S.aureus* were obtained from the microbial bank of Antimicrobial Resistance Research Center (ARRC) of Iran University of Medical Sciences, Tehran, Iran. All strains were collected from inpatients with burn injuries and conserved in tryptic soy broth (TSB) with 20% glycerol. An MRSA ATCC 33591 got along with the rest. *Staphylococcus aureus *ATCC 25922 was used as the quality control. The strains were recultured from TSB on blood agar and incubated for 18 hours, for further analyses. 

**Antibiotics**

Ten types of antibiotics from different classes routinely used in treatment of staphylococcal infections were selected, including cefazolin, rifampicin, vancomycin, oxacillin, clindamycin, cotrimoxazole, minocycline, linezolid, azithromycin, and clarithromycin. It should be noted that various types of antimicrobial agents are used against staphylococcal infections. In the current study, the tested antibiotics were selected from the most therapeutic options especially in Iran. The disks and powders of the antibiotics were purchased from MAST (UK) and Sigma Aldrich (4), respectively.

**Identification tests**

Isolates were confirmed by their Gram-positive, catalase positive, DNase positive reaction, mannitol fermentation activity, and coagulase positive results (13).

**Detection of MRSA strains**

Disk diffusion test to evaluate the resistance to oxacillin and cefoxitin was done as described in Clinical and Laboratory Standards Institute (CLSI) guidelines(14), focused on cefoxitin, which gives more accurate result than oxacillin.

**Detection of biofilm producing** ***Staphylococcus aureus***

Qualitative detection of biofilm-producer strains was performed by culturing 28 strains on Congo red agar (CRA)(15). This method is based on the morphological characteristics of biofilm-producer and non-producer bacteria cultured on Congo red medium, composed of brain heart infusion broth (BHI) 37g/L, sucrose 50 g/L, agar No.1 10 g/L, and Congo red 0.8 g/L. Congo red stain was prepared as a concentrated aqueous solution and sterilized at 121˚C for 15 minutes. Then, it was added to the rest of autoclaved medium. The strains were inoculated in streaks and incubated at 35°C for 24 and 48 hours, aerobically. Black colonies with a dry crystalline consistency indicated biofilm production, while the non-biofilm-producing strains formed red colonies(16). The quantitative detection of biofilm production of each strain, carried out by microtiter plate biofilm assay, described below.

**Determination of minimum inhibitory concentration value**

MIC assays were performed against 10 antimicrobial agents in Mueller-Hinton broth(MHB) according to CLSI guidelines for broth microdilution susceptibility test(14).Strains were incubated at 37°C for 20 to 24 hours, and susceptibility of the isolates was determined according to the standard CLSI breakpoints. The MIC is determined as the lowest concentration of an antimicrobial agent at which the bacterial growth is completely inhibited.

**Microtiter plate biofilm assay, as a quantitative biofilm production assay**

The impact of 10 antibiotics was assessed on biofilm formation in subinhibitory concentrations as previously described (2,17,18). Briefly, aliquots (100 µL) of antibiotic solutions (512 µg/mL) were added in triplicate to100 µL tryptic soy broth (TSB) supplemented with 1% glucose in the first well of a 96-well tissue culture microtiter plate (Falcon). The serial dilutions were provided with the concentrations ranged from 0.06to 128 µg/ml(serial dilution: 0.06, 0.125, 0.5, 1, 2, 4, 8, 16, 32, 64, 128 µg/mL) and wells containing uninoculated TSB+glucose 1% served as a negative controls. Aliquots of inocula (100 µLeach, ca. 10^4^ to 10^5 ^CFU/ml) were added to the wells. Positive controls were prepared by mixing 100 µL of inocula and 100 µLofTSB+glucose1%without antibiotics. The plates were covered and incubated at 37°C for 24 hours.

The content of the wells were aspirated and the microtiter plates were washed twice with sterile normal saline to eliminate unbound cells, and then, dried. To fix the attached cells, 190 µL of methanol (99%) was added to each well, left for 20 minutes, and then, decanted and dried. Afterward, the adherent cells were stained for 5 minutes with 190 µL of Gram’s crystal violet. The wells were then, rinsed with tap water 3 times to rinse the excess stain off. Plates were air-dried at room temperature. To quantitate the biofilm biomass, the dye bound to the attached cells were released by adding 190 µL of glacial acetic acid 33% v/v to the wells, and the plates were placed in ELISA (the enzyme-linked immunosorbent assay) reader after shaking gently for 30 seconds to measure the absorbance at 630 nm wavelength. The percentage of biofilm inhibition is expressed as [(OD_630 _value at0µg/mL of antibiotic −[ OD_630_ value in the presence of an antibiotic) / OD_630 _value at 0 µg/mL of antibiotic ] × 100 (18). The positive values showed inhibitory effect, and negative values represent inducing effect of antibiotics on biofilm formation according to the formula.

**Statistical analysis**

The significance of differences between the mean of optical densities of groups, with and without each antibiotic (12 groups; the serial dilutionranged from 0.06to128 µg/mL) were examined by one-way analysis of variance (ANOVA), followed by the Dunnett multiple comparison test. A P-value of <0.05 was considered statistically significant. All calculations were carried out using Graph Pad prism 6.

## Results

**Strain selection**

**Figure 1:**
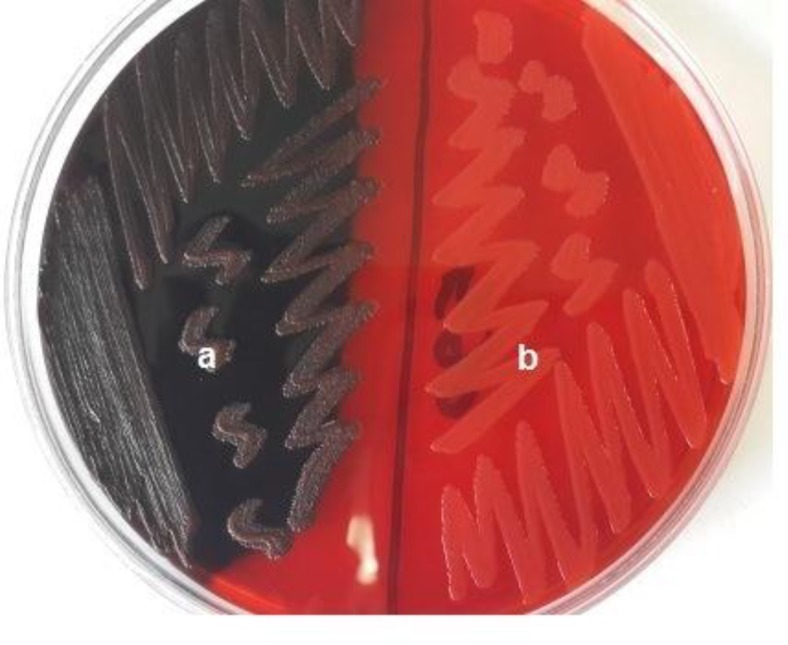
Colony colors on CRA;(A) Black colonies, biofilm producers; (B) Red colonies, non-biofilm producers

Two conditions were considered to select the appropriate strains for further studies: the ability to produce biofilm, and showing resistance to various antibiotics. Among the 28 strains of *S.aureus* isolated from clinical samples, 5strains were qualified on the basis of biofilm production by the Congo red agar (CRA) method ;the strains with black colonies were considered as biofilm-producer (figure 1), and high resistance to antibiotics used in the current study.

The strains disqualified for different reasons such as low biofilm-forming ability or high sensitivity to the mentioned antibiotics. One MSSA and 3 MRSA strains were detected by the disk diffusion method for resistance to oxacillin and cefoxitin. MRSA ATCC 33591 was used along with these 4clinical strains.

**MIC determination**

MIC values of 5 isolates against cefazolin, rifampicin, vancomycin, oxacillin, clindamycin, cotrimoxazole, minocycline, linezolid, azithromycin, and clarithromycin are shown in Table 1.

**Table 1:** MICs of the Antibiotics Tested in the Current Study

Strain	MIC (µg/mL)									
	CAZ	AZM	VAN	OXA	MIN	RIF	CLI	TMP/SMX	LZD	CLR
*S.aureus*633	128	128	0.5	128	128	0.06	128	21.3/106.7	128	128
*S.aureus*595	128	128	0.5	128	128	128	128	21.3/106.7	128	128
*S.aureus*622	4	128	1	0. 5	128	0.06	8	0.6/3.3	8	128
*S.aureus*627	128	128	0.5	128	128	0.06	64	21.3/106.7	128	128
*MRSA33591*	128	128	1	32	128	0.06	128	2.6/13.3	64	128

MIC, minimuminhibitory concentration; CAZ, cefazolin; AZM, azithromycin; VAN, vancomycin; OXA, oxacillin; MIN, minocycline; RIF, rifampin; CLI, clindamycin; TMP/SMX, trimethoprim-sulfamethoxazole; LZD, linezolid; CLR, clarithromycin

Of the studied isolates, all the 5isolates were resistant to azithromycin, clarithromycin, minocycline, clindamycin and linezolid, and 1strain (*S.aureus* 595) was resistant to rifampicin, whereas all the strains were susceptible to vancomycin. Four of the five isolates, were resistant to cefazolin, oxacillin, and cotrimoxazole. These resistance patterns can help to ignore the direct impact of antibacterial activity of agents on the growth, and subsequently, the biofilm production of strains.

**Influence of sub-MICs on biofilm formation**

By means of the microtiter plate biofilm assay, isolates were incubated in the presence of antimicrobial concentrations ranging from 1× to 1/1024 MIC (these concentrations are below the MICs of the strains accounted for sub-minimal inhibitory concentration).These MIC ratios, which refer to continuous dilutions of antibiotics, are different for each strains depending on their MIC, and it should be noted that for the strains, which showed the MIC of over 128 µg/mL, the ratios of MIC were defined by considering the least sum of MIC(128 µg/mL). The percentages of biofilm inhibition or induction are shown in Table 2.

**Table 2:** Inhibition of Biofilm Formation of *Staphylococcus aureus* Isolates at Six Sub-MICs of the Tested Antibiotics

			% Inhibition of Biofilm Formation											
Isolate	MIC(µg/mL)	1/2MIC		1/4MIC	1/8MIC			1/16MIC		1/32MIC				1/64MIC
					**Cefazolin**									
*S.aureus*633	128	71^*a^		60*	45*			57*		54*				51*
*S.aureus*595	128	66*		56*	53*			46*		60*				83*
*S.aureus*622	4	14		14	7			-14		7				-7
*S.aureus*627	128	66*		43*	16			36*		10				33*
*MRSA 33591*	128	53*		25	-8			-6		-16				-28
	**Azithromycin**													
*S.aureus*633	128	7		-17	-32			-22		12				0
*S.aureus*595	128	3		-3	0			3		3				16
*S.aureus*622	128	6		-6	-66*			-6		0				0
*S.aureus*627	128	26		20	-6			-23		-76*				-70*
*MRSA 33591*	128	3		-21	-28*			-57*		-68*				-70*
	**Vancomycin**													
*S.aureus*633	0.5	-80*		-48*	-84*			-b		-				-
*S.aureus*595	0.5	0		0	-52*			-		-				-
*S.aureus*622	1	45*		48*	54*			57*		-				-
*S.aureus*627	0.5	6		-12	-38*			-		-				-
*MRSA 33591*	1	17		-4	-19			-23		-				-
	**Oxacillin**													
*S.aureus*633	128	45*		33*		29		37*		41*				33*
*S.aureus*595	128	37*		23*		30*		35*		32*				32*
*S.aureus*622	0.5	31		-26		-52*		-		-				-
*S.aureus*627	128	39*		34*		19		26		36*				41*
*MRSA 33591*	32	-10		2		-7		15		-66*				-89*
						**Minocycline**								
*S.aureus*633	64	-11		-11		-27		-22		5				-11
*S.aureus*595	64	8		-22		-34		-28*		-14				-68*
*S.aureus*622	64	50*		42*		42*		39*		39*				39*
*S.aureus*627	64	48*		25		13		18		10				15
*MRSA 33591*	64	66*		42*		44*		27*		17				21
		% Inhibition of Biofilm Formation													
Isolate	MIC(µg/mL)	1/2MIC		1/4MIC	1/8MIC			1/16MIC		1/32MIC			1/64MIC	
	**Cotrimoxazole**													
*S.aureus*633	21.3/106.7	-2		-5	5			-4		20			5	
*S.aureus*595	21.3/106.7	30		-2	-23			-15		-2			-38*	
*S.aureus*622	0.6/3.3	-49*		-52*	-50*			-18		-21			-29*	
*S.aureus*627	21.3/106.7	37*		18	18			9		-20			-2	
*MRSA 33591*	2.6/13.3	57*		23	9			-7		-19			-3	
	**Rifampin** c													
*S.aureus*633	0.06				-									
*S.aureus*595	128	42*		51*	15			0		21			-6	
*S.aureus*622	0.06				-									
*S.aureus*627	0.06				-									
*MRSA 33591*	0.06				-									
	**Clindamycin**													
*S.aureus*633	128	50*		46*	35*			29*		24			20	
*S.aureus*595	128	34*		42*	40*			34*		25			42*	
*S.aureus*622	8	27		9	18			27		18			9	
*S.aureus*627	64	15		31	50*			33		30			49*	
*MRSA 33591*	128	-83*		-88*	0			-9		-19			-23	
	**Linezolid**													
*S.aureus*633	128	37*		41*		37*	22				22		50*	
*S.aureus*595	128	25		50*		0	25				0		0	
*S.aureus*622	8	16		28*		14	-4				4		16	
*S.aureus*627	128	13		41*		20	30*				24		26	
*MRSA 33591*	64	54*		52*		42*	42*				38*		38*	
	**Clarithromycin**													
*S.aureus*633	128	32*		40*	32*		29*				25*		-14	
*S.aureus*595	128	45*		44*	31*		22				22		11	
*S.aureus*622	128	15		10	-15		-5				-15		-10	
*S.aureus*627	128	73*		65*	59*		30*				39*		33*	
*MRSA 33591*	128	11		-5	14		3				-11		-15	

ATCC, American type culture collection

a* : P<0.05

b ^: ^The ratio of MIC mentioned in the table depends on the MIC of each strain. Some concentrations were excluded because they were not in the range of the tested area (0.06to 128 µg/mL).

c ^:^Asthe range of testing concentrations was started from 0.06 µg/mL, and the sub-MICs of 4 strains for rifampin were less than this concentration, they were not included in this table.

Since most of the significant effects (P<0.05) were observed in the range of 1/2 to 1/64 MIC, this range was considered in the mentioned table and other concentrations were not shown. The lowest effect was observed when linezolid was applied in all 5 isolates in the presence of at least 2subinhibitory concentrations, which had significant inhibiting effect on biofilm formation(P<0.05). Afterward, cefazolin (in 4strains) and clarithromycin, oxacillin, clindamycin, and minocycline had significant reducing effects on the biofilm formation of 3isolates, followed by cotrimoxazole (2strains) and vancomycin (1strain).

In contrast, some antibiotics showed inducing effects on biofilm formation. For instance, azithromycin and vancomycin had great inducing impacts on the biofilm growth of 3 strains, in the presence of at least 1subinhibitory concentration, followed by oxacillin and cotrimoxazole in 2strains, and 1 strain, respectively, when minocycline and clindamycin were used. The number of strains, which induced, reduced, and affected by the antibiotics is shown in Table 3.

**Table3:** The Effect of Sub-minimal Inhibitory Concentrations of Antibiotics on the Level of *Staphylococcus aureus* Biofilm Formation

Antimicrobial Agent	No. of Isolates/ Total No. of Tested Isolates		
	Increase Decrease No effect		
Linezolid	0/5	5/5	0/5
Oxacillin	2/5	3/5	0/5
Cefazolin	0/5	4/5	1/5
Vancomycin	3/5	1/5	1/5
Minocycline	1/5	3/5	1/5
Cotrimoxazole	2/5	2/5	1/5
Clindamycin	1/5	3/5	1/5
Clarithromycin	0/5	3/5	2/5
Azithromycin	3/5	0/5	2/5
Rifampicin	0/5	1/5	4/5

Table 3 is a simplified form of Table2; for more information, refer to the previous table.

The maximum effects were detected at 1/2 and 1/4 MICs, followed by 1/8 MIC in most of the antibiotics, except azithromycin. For the last antibiotic, in 3isolates, the inducing effect was observed at 1/8 to 1/64 MICs.

## Discussion

Typically, the doses of antibiotics prescribed by physicians are on the basis of MIC of the pathogen causing the infection, as it is reasonably expected to be equal or higher than that of the MICs effective in the elimination of infections. In contrast, recent studies ascertained that sub-minimal inhibitory concentrations of antibiotics had strong influence on the biofilm formation process. The ability of biofilm formation is an important virulence factor, particularly in MRSA implant-associated infections in humans. The structure of biofilm has a high resistance in comparison with planktonic cells. The results of several studies demonstrated that the significant reducing and inducing effects on biofilm formation rate are dependent on the antimicrobial agents and even vary from one strain to another. Different mechanisms are defined for the impact of low doses of antibiotics on the biofilm production, as quorum sensing inhibition and the impact on the expression of intercellular adhesion gene cluster (15). The exposure of bacteria to sub-MIC antibiotics may commonly occur during the normal course of antibiotic therapy. The results of the present study demonstrated that sub-MIC of therapeutic antibiotics, such as azithromycin and vancomycin, could significantly induce the biofilm formation in at least 2 isolates of *S.aureus*, which may cause adverse effects on the course of treatment. This result appears to contradict some previous studies in this field and also concur with some others at the same time. It should be mentioned that different strains used in various tests may cause different results. For this reason, further studies seem necessary in this area. Despite the numerous studies on antibiotics that affected the biofilm-production of Gram-negative bacteria, there is contradictory evidence about these effects on Gram-positive ones(19).In contrast to the abovementioned results of vancomycin and azithromycin; cefazolin, linezolid, oxacillin, clindamycin, minocycline, and clarithromycin showed significant inhibitory effects on biofilm roduction in lower concentrations than the MICs of strains, which are possibly considered as effective drugs on biofilms and infections, subsequently. These 10antibiotics were selected for the study because of high usage in prescription for therapeutic purposes, especially for *S.aureus*.

 The sub-MIC effects of different antibiotics on pathogenic factors are tested by many investigators in the recent years. The inducing effects of some agents applied in the treatment of infections should be considered more, as a serious challenge by doctors. Besides, the types of impact of an antibiotic can be different, even strain to strain. This issue may occur when an antibiotic has binary effect on unlike organisms used in vivo*. *In such cases, the effective concentrations can reduce the biofilm formation of a microorganism and induce other organisms simultaneously. Except *S.epidermidis, *a few studies were conducted on other staphylococci. In terms of *S.epidermidis*, Gomes et al., observed no significant reduction in biofilm-formation ability with 8commonly used antibiotics to treat Gram-positive infections(20).Mirani and jamil showed that the sub-MICs of vancomycin and oxacillin promoted *S.aureus* biofilm formation on nylon and silicon surfaces(21). Some studies focused on anti-biofilm activity of macrolides (such as azithromycin, clarithromycin, and erythromycin) against Gram-negative organism, but there is contradictory information about the impacts of macrolides on biofilm-formation of Gram-positive organisms that the current study results were in agreement with. Rachid et al., reported that erythromycin can stimulate the expression of *ica* gene(22). But, Parra-Ruiz et al., showed that low levels of clarithromycin could inhibit the biofilm formation process of *S.aureus*(19). Other studies reported an inhibitory or no effects of other antibiotics used in the current study such as vancomycin(19), clarithromycin, and azithromycin(23) by low concentrations on *S.aureus *biofilm-formation. Nevertheless, inconsistent results obtained from different studies may happen because the sub-MIC effects can vary from one strain to another. Additionally, most of the studies tested antibiotics at single or limited concentrations(2).Dunne showed that the biofilm ODr (OD ratio) increased when growing biofilms were incubated with subinhibitory concentrations of vancomycin and cefamandole, indicating that biofilm production is a sort of defense reaction of the bacteria(23).Jeffrey B. Kaplan et al., showed that low doses of β-lactam antibiotics induced extracellular DNA release and biofilm-formation in *S. aureus*(2). Boles and Horswill reported that sub-MICs of cefalotin induced *S. aureus *biofilm-formation, but did not have an impact on the expression of *agr*, a quorum sensing system that modulates *S. aureus *biofilm-formation and dispersal(24). In the current study, the utilized concentration range was effectively extensive (1× to 1/1024 MIC), and tried to include an appropriate number of antibiotics commonly used in the therapies.

## Conclusion

Unlike some previous studies, the current study findings showed that some therapeutic antibiotics, such as azithromycin and vancomycin, may have reversal effects on the treatment of infections caused by MRSA, by increasing the level of biofilm formation, which is an essential pathogenic factor. In contrast, linezolid, cefazolin, and clarithromycin, and in the second place, clindamycin and minocycline could be proper choices to inhibit the biofilm formation of *S.aureus. *

The in vitro and in vivo studies are numerous enough to reconsider the dosing designs of the widely used antibiotics to treatstaphylococcal infections.
